# Immunotherapy and Relevant Antibody–Drug Conjugates in Gynecologic Oncology: Recent Advances, Ongoing Challenges, and Future Directions

**DOI:** 10.3390/cancers18142342

**Published:** 2026-07-20

**Authors:** Ting-Tai Yen, Tina Yi-Jin Hsieh, Eugene P. Toy

**Affiliations:** 1 Department of Obstetrics and Gynecology, Texas Tech University Health Sciences Center El Paso, El Paso, TX 79905, USA; 2 Department of Obstetrics and Gynecology, Beth Israel Deaconess Medical Center, Boston, MA 02215, USA; 3 Division of Gynecologic Oncology, Department of Obstetrics and Gynecology, Texas Tech University Health Sciences Center El Paso, El Paso, TX 79905, USA

**Keywords:** gynecologic cancer, immunotherapy, immune checkpoint inhibitor, antibody–drug conjugate, endometrial cancer, cervical cancer, ovarian cancer, biomarkers, programmed cell death protein 1 (PD-1), human epidermal growth factor receptor 2 (HER2)

## Abstract

Immune checkpoint inhibitors and antibody–drug conjugates have rapidly expanded treatment options for gynecologic malignancies, although the magnitude of benefit varies substantially across tumor types and biomarker-defined populations. This narrative review summarizes the biologic rationale, predictive biomarkers, pivotal clinical trials, regulatory approvals, guideline-supported strategies, and emerging directions for immune checkpoint blockade and antibody–drug conjugates in endometrial, cervical, and ovarian cancers. In endometrial cancer, molecular classification and mismatch repair status have transformed treatment selection, with PD-1 or PD-L1 blockade now integrated into first-line chemoimmunotherapy and recurrent disease management. HER2-directed and TROP-2-directed antibody–drug conjugates are also emerging as biomarker-directed strategies. In cervical cancer, human papillomavirus-driven tumor biology, PD-L1 expression, and tissue factor expression support the use of checkpoint inhibitors, antibody–drug conjugates, and therapeutic vaccine approaches across locally advanced and recurrent or metastatic settings. In ovarian cancer, single-agent checkpoint blockade has shown limited activity in unselected populations, but recent advances include biomarker-selected chemoimmunotherapy in platinum-resistant disease and clinically meaningful activity of folate receptor alpha-directed and HER2-directed antibody–drug conjugates. Across gynecologic cancers, key challenges include refining predictive biomarkers, optimizing sequencing after prior immunotherapy exposure, managing overlapping toxicities, and designing trials that enrich for biologically responsive subgroups. Future progress will depend on integrating molecular classification, immune contexture, ADC target expression, and patient-specific clinical factors into treatment selection.

## Simple Summary

Gynecologic cancers, including endometrial, cervical, and ovarian cancers, remain major causes of illness and death worldwide. New treatments are changing how these cancers are managed. Immune checkpoint inhibitors help the immune system recognize and attack cancer cells, while antibody–drug conjugates deliver cancer-killing drugs more directly to tumor cells. These treatments have been especially important for selected patients whose tumors have specific characteristics, such as mismatch repair deficiency, programmed death-ligand 1 (PD-L1) expression, or human epidermal growth factor receptor 2 (HER2) overexpression or amplification. This review summarizes how these treatments work, how biomarkers may guide patient selection, and how recent clinical trials have shaped treatment options. We also discuss ongoing challenges, including treatment sequencing, resistance, and the need for better biomarker-based clinical trials.

## 1. Introduction

Gynecologic malignancies, including ovarian, endometrial, and cervical cancers, represent a substantial global health burden, accounting for approximately 1.4 million new cases and 650,000 deaths annually worldwide [1]. Despite advances in surgery, chemotherapy, radiation, antiangiogenic therapy, and targeted treatment, many patients with advanced or recurrent gynecologic cancers continue to experience disease progression, treatment resistance, cumulative toxicity, and poor long-term outcomes. These challenges are particularly evident in advanced ovarian cancer, where most patients ultimately relapse after platinum-based therapy; in endometrial cancer, where incidence and mortality continue to rise; and in cervical cancer, which remains a major cause of cancer mortality worldwide, especially in low-resource settings [2,3,4,5].

The expanding molecular characterization of gynecologic cancers has created new opportunities for biomarker-directed therapy. Immune checkpoint inhibitors (ICIs), particularly agents targeting the programmed cell death protein 1/programmed death-ligand 1 (PD-1/PD-L1) axis, have transformed treatment for selected populations, most notably mismatch repair-deficient (dMMR) or microsatellite instability-high (MSI-H) endometrial cancers and PD-L1-positive cervical cancers [6,7,8,9]. In parallel, antibody–drug conjugates (ADCs) have emerged as an important therapeutic class by delivering potent cytotoxic payloads to tumors expressing clinically actionable surface targets, including tissue factor, folate receptor alpha (FRα), and human epidermal growth factor receptor 2 (HER2) [10,11,12]. Together, ICIs and ADCs reflect a broader shift from empiric cytotoxic therapy toward biologically guided treatment selection.

This review summarizes the evolving role of ICIs and ADCs in endometrial, cervical, and ovarian cancers. We highlight key biologic rationales, predictive biomarkers, pivotal clinical trials, approved and guideline-supported treatment strategies, emerging combinations, current challenges, and future directions.

## 2. Mechanistic Framework for ICIs and ADCs

### 2.1. Immune Checkpoint Biology and Biomarkers of Response

Antitumor immunity depends on tumor antigen capture and presentation by antigen-presenting cells, particularly dendritic cells, followed by activation of tumor-specific T cells through T-cell receptor recognition and costimulatory signaling. Immune checkpoints are physiologic inhibitory pathways that prevent excessive immune activation and autoimmunity; however, tumors can exploit these pathways by upregulating checkpoint ligands and shaping an immunosuppressive tumor microenvironment. ICIs restore antitumor immune activity by blocking these inhibitory signals rather than directly killing tumor cells [13,14,15,16].

The most established checkpoint targets in gynecologic oncology are PD-1, PD-L1, and CTLA-4. PD-1/PD-L1 blockade primarily reinvigorates pre-existing antitumor T-cell activity within the tumor microenvironment [17], whereas CTLA-4 blockade acts earlier during T-cell priming and may broaden immune activation [13,17,18,19]. Due to its mechanistically complementary activity at distinct stages of the immune response, dual CTLA-4/PD-1 blockade provides a strong biologic rationale for continued investigation across gynecologic cancer subtypes, particularly in histologies where single-agent ICI efficacy remains limited [20,21,22].

Several biomarkers help explain differential ICI responsiveness across gynecologic cancers. Mismatch repair deficiency (dMMR) and microsatellite instability-high (MSI-H) status are associated with higher mutation burden, making these tumors more immunogenic and more likely to respond to checkpoint blockade [23,24]. High tumor mutational burden (TMB) may similarly increase neoantigen presentation and ICI sensitivity [25]. PD-L1 expression reflects an adaptive immune resistance mechanism [26,27,28] and has been well established as a treatment-selection biomarker in cervical cancer, where even a low threshold of PD-L1 CPS ≥1 has guided the use of pembrolizumab-based therapy in recurrent, persistent, and/or metastatic disease [9,29,30]. However, PD-L1 alone is an imperfect biomarker, and ICI response is also shaped by tumor antigenicity, T-cell infiltration, antigen presentation, myeloid suppression, angiogenesis, and the spatial organization of immune cells within the tumor microenvironment [31,32].

Beyond PD-1/PD-L1 and CTLA-4, several emerging checkpoint pathways are under investigation in gynecologic malignancies, including both co-inhibitory receptors and co-stimulatory pathways [33,34]. Among co-inhibitory receptors, lymphocyte activation gene 3 (LAG-3) suppresses T-cell activation, proliferation, cytokine secretion, and cytotoxic function [17]. In ovarian cancer, LAG-3 can be co-expressed with PD-1 on dysfunctional tumor-infiltrating CD8+ T cells, supporting the rationale for dual LAG-3 and PD-1 blockade [35,36,37]. Other co-inhibitory receptors, including T-cell immunoglobulin and mucin domain containing–3 (TIM-3) and T-cell immunoreceptor with immunoglobulin and ITIM domains (TIGIT), have also been implicated in T-cell exhaustion and resistance to PD-1/PD-L1 blockade. TIM-3 is frequently co-expressed with PD-1 on exhausted T cells and is often considered a marker of deeper T-cell dysfunction or resistance to PD-1 blockade [17,38]. TIGIT suppresses T cells, natural killer cells, and antigen-presenting cells through the TIGIT–CD155–CD226 axis, thereby weakening costimulatory signaling [38]. Both TIM-3 and TIGIT have been investigated as potential partners for PD-1/PD-L1 blockade in gynecologic cancers, with the goal of overcoming adaptive immune resistance [39]. In contrast, co-stimulatory receptors including OX40, CD40, and 4-1BB (CD137) function as positive regulators of T-cell activation and augment immunological responses against malignant cells [40]. Together, these emerging co-inhibitory and co-stimulatory checkpoints provide a biologic rationale for combination strategies designed to overcome immune resistance in gynecologic cancers [17,33,41].

### 2.2. ADCs and Biomarker-Directed Cytotoxic Delivery

ADCs are targeted therapeutic platforms that combine the antigen specificity of monoclonal antibodies with the cytotoxic potency of chemotherapy. Structurally, ADCs consist of three key components: an antibody directed against a tumor-associated surface antigen, a chemical linker, and a cytotoxic payload [42,43,44]. After antigen binding, the ADC–antigen complex is internalized by the tumor cell and releases its payload intracellularly. The released payload induces tumor cell death through mechanisms such as DNA damage, mitotic arrest, or other forms of lethal cellular injury [43,44].

ADC efficacy is influenced by linker stability, payload properties, and drug-to-antibody ratio (DAR). Linker design influences where and how efficiently the cytotoxic payload is released. Cleavable linkers, used in agents such as tisotumab vedotin, mirvetuximab soravtansine, sacituzumab govitecan, and trastuzumab deruxtecan, can facilitate intracellular payload release and may enable bystander killing when the released payload is membrane-permeable and capable of diffusing into neighboring antigen-low or antigen-negative tumor cells. In contrast, non-cleavable linkers, such as the linker used in trastuzumab emtansine (T-DM1), generally limit payload activity to antigen-expressing target cells, thereby reducing bystander effects [43,45,46]. Linker stability is critical because premature payload release in systemic circulation can reduce tumor-directed drug delivery and expose normal tissues to cytotoxic payloads, thereby increasing off-target toxicity [47]. Payload class further shapes ADC activity: microtubule inhibitors disrupt mitosis, whereas topoisomerase I inhibitor payloads, including deruxtecan/DXd and SN-38, induce replication-associated DNA damage and may enhance bystander activity because of their membrane permeability [48,49,50]. Finally, DAR influences potency, pharmacokinetics, and toxicity. Although a higher DAR can increase payload delivery, excessive drug loading may impair solubility, alter clearance and tissue distribution, and narrow the therapeutic window [49,51].

In gynecologic oncology, clinically relevant ADC targets include folate receptor alpha (FRα) in ovarian cancer, tissue factor in cervical cancer, and HER2 across selected gynecologic tumors. FRα-directed therapy is exemplified by mirvetuximab soravtansine in FRα-positive platinum-resistant ovarian cancer [11,52], whereas tissue factor is targeted by tisotumab vedotin in recurrent or metastatic cervical cancer [10,53]. HER2-directed ADC therapy, particularly trastuzumab deruxtecan, has shown activity across selected HER2-expressing solid tumors, including endometrial, cervical, and ovarian cancer cohorts in DESTINY-PanTumor02 [12]. Biomarker testing is therefore central to ADC treatment selection. Disease-specific ADC biomarkers and testing considerations are discussed in the endometrial, cervical, and ovarian cancer sections below.

## 3. Endometrial Cancer

Endometrial cancer (EC) has become the gynecologic malignancy most transformed by immunotherapy, driven by its molecular heterogeneity and the strong predictive value of mismatch repair and mutational status for immune checkpoint inhibitor response. Current guidelines, including NCCN and ESGO, now emphasize molecular profiling to guide risk stratification, adjuvant treatment selection, and systemic therapy decisions in advanced or recurrent disease [3,54,55].

### 3.1. Molecular Classification and Immunologic Rationale

Endometrial cancer classification has shifted from reliance on histopathology alone toward objective molecular profiling, because molecular features more accurately convey prognosis, guide therapy selection, and inform biomarker-driven trial design [56]. The Cancer Genome Atlas first defined four molecular groups, namely POLE ultramutated, microsatellite-instability hypermutated (MSI-H), copy-number low, and copy-number high tumors, establishing a genomic framework that now underpins modern management [57]. This framework has been translated into clinically deployable classification using pathogenic POLE mutation testing, mismatch repair deficiency by immunohistochemistry (IHC) or MSI testing, p53 status by IHC or sequencing, and classification of tumors without these features as no specific molecular profile (NSMP) [56].

The biology of POLE-mutant and MMRd/MSI-H tumors, characterized by high mutational and neoantigen burden, provides a strong rationale for immune checkpoint blockade [58]. MMR deficiency is associated with MSI-H status and increased tumor mutational burden (TMB), making MMR/MSI status a central biomarker for treatment selection [3,23,24]. Together, these molecular features support the use of ICIs in biomarker-selected endometrial cancer and reinforce the importance of universal molecular testing at diagnosis.

### 3.2. First-Line Chemoimmunotherapy for Advanced or Recurrent EC

**Figure 1:**
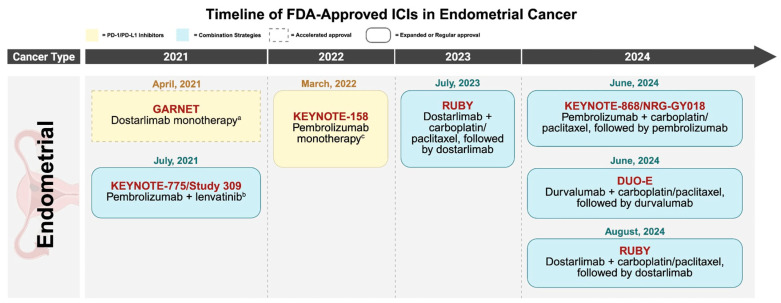
Timeline of FDA approvals of ICIs in endometrial cancer. Abbreviations: FDA, U.S. Food and Drug Administration; ICIs, immune checkpoint inhibitors. ^a^ Dostarlimab monotherapy received accelerated FDA approval in April 2021 and regular approval in February 2023 for dMMR recurrent or advanced EC after progression on or following platinum-containing therapy. ^b^ Pembrolizumab plus lenvatinib initially received accelerated FDA approval in 2019 for previously treated advanced EC that was not MSI-H/dMMR and received regular approval in July 2021. ^c^ Pembrolizumab monotherapy was FDA-approved in March 2022 for advanced MSI-H or dMMR EC after prior systemic therapy, based on KEYNOTE-158.

In the first-line advanced or recurrent setting, several phase III trials have established immune checkpoint blockade combined with carboplatin and paclitaxel as a major treatment strategy. In RUBY, dostarlimab plus carboplatin and paclitaxel significantly prolonged progression-free survival (PFS), with the greatest benefit observed in the dMMR/MSI-H subgroup [8]. Similarly, NRG-GY018 demonstrated that pembrolizumab plus carboplatin and paclitaxel improved PFS in both dMMR and pMMR cohorts, with a particularly pronounced benefit in dMMR disease [7,59]. DUO-E evaluated durvalumab with carboplatin and paclitaxel followed by durvalumab maintenance, with or without olaparib, and demonstrated statistically significant and clinically meaningful PFS improvement with durvalumab-based strategies [60]. AtTEnd/ENGOT-en7 evaluated atezolizumab with carboplatin and paclitaxel and showed a PFS benefit that was most pronounced in dMMR disease [61] (Figure 1).

Taken together, these first-line trials establish chemoimmunotherapy as a new standard-of-care framework for advanced or recurrent EC. The magnitude of benefit is greatest and most consistent in dMMR/MSI-H tumors. In this evolving first-line chemoimmunotherapy landscape, U.S. FDA-approved ICI-based options for patients with primary advanced or recurrent EC now include pembrolizumab plus carboplatin and paclitaxel followed by pembrolizumab maintenance, dostarlimab plus carboplatin and paclitaxel followed by dostarlimab maintenance, and, for dMMR disease, durvalumab plus carboplatin and paclitaxel followed by durvalumab maintenance.

No prospective head-to-head trial has directly compared pembrolizumab-, dostarlimab-, and durvalumab-based chemoimmunotherapy in advanced or recurrent EC; therefore, cross-trial comparisons should be interpreted cautiously. In current practice, selection among ICI-based regimens is guided by MMR/MSI status, histology, regulatory indication, trial eligibility, maintenance strategy, toxicity profile, and institutional experience rather than proven superiority of one checkpoint inhibitor over another. A recent clinical commentary proposed a flow diagram to guide upfront treatment selection [62]. Notably, 4-year follow-up data from RUBY demonstrated sustained remission and survival with dostarlimab plus carboplatin/paclitaxel in dMMR/MSI-H advanced or recurrent EC, despite the fact that some patients in the placebo arm later crossed over to receive dostarlimab [63]. These long-term data support dostarlimab-based chemoimmunotherapy as a particularly well-supported frontline option in dMMR/MSI-H disease, while direct comparative efficacy among ICI-based regimens remains undefined.

### 3.3. Biomarker-Selected Therapy After Prior Treatment

For previously treated recurrent disease, biomarker-selected single-agent PD-1 blockade remains an important strategy for MSI-H/dMMR tumors, particularly in patients who have not previously received an immune checkpoint inhibitor. Pembrolizumab demonstrated antitumor activity in patients with previously treated MSI-H/dMMR advanced EC in KEYNOTE-158 and is FDA approved as monotherapy for advanced MSI-H/dMMR EC following prior systemic therapy [6,64]. Dostarlimab also demonstrated durable responses in dMMR recurrent or advanced EC in GARNET, with an ORR of 45.5% in the dMMR/MSI-H EC cohort [65]. FDA labeling supports dostarlimab monotherapy for dMMR recurrent or advanced endometrial cancer after progression on or following platinum-containing therapy.

In contrast, MMR-proficient/microsatellite-stable (pMMR/MSS) tumors have limited sensitivity to single-agent PD-1 blockade, and combination strategies are generally needed to achieve meaningful activity. Pembrolizumab plus lenvatinib is the key established post-platinum regimen in this setting. In KEYNOTE-775/Study 309, pembrolizumab plus lenvatinib significantly improved PFS and overall survival (OS) compared with chemotherapy in previously treated advanced EC, including in the pMMR subgroup [66]. This regimen is FDA-approved for advanced EC that is pMMR or not MSI-H after prior systemic therapy.

These combined data support a biomarker-directed approach to recurrent EC after prior therapy. For patients with dMMR/MSI-H tumors who are ICI-naïve, single-agent PD-1 blockade can provide durable responses. In contrast, pMMR/MSS tumors generally require combination therapy, with pembrolizumab plus lenvatinib remaining an established post-platinum option.

### 3.4. HER2-Directed and Other ADC-Based Strategies

Molecularly targeted strategies are also advancing in EC, particularly in HER2-positive disease. In HER2-positive EC, especially uterine serous carcinoma and carcinosarcoma, trastuzumab improves outcomes when added to carboplatin and paclitaxel [67]. More recently, the HER2-directed ADC trastuzumab deruxtecan (T-DXd) has shown clinically meaningful activity across HER2-expressing solid tumors, with the greatest benefit in IHC 3+ disease, in DESTINY-PanTumor02 [12]. Based on pan-tumor data, the FDA granted accelerated tumor-agnostic approval to T-DXd for previously treated unresectable or metastatic HER2-positive IHC 3+ solid tumors when no satisfactory alternative treatment options are available. For endometrial cancer specifically, NCCN-listed use is broader than the FDA tumor-agnostic label, which includes HER2-positive tumors with IHC 3+ or 2+ expression in the recurrent disease setting, whereas the FDA tumor-agnostic approval is limited to IHC 3+ solid tumors.

Another ADC under investigation in EC is sacituzumab govitecan, a trophoblast cell surface antigen 2 (TROP-2)-directed ADC. In TROPiCS-03, sacituzumab govitecan demonstrated encouraging activity in advanced EC after prior platinum-based therapy and immunotherapy [68]. Building on these data, ASCENT-GYN-01/GOG-3104/ENGOT-en26/APGOT-EN2 was developed to compare sacituzumab govitecan with physician’s choice chemotherapy in recurrent or persistent EC after platinum-based chemotherapy and PD-1/PD-L1 inhibitor therapy, with dual primary endpoints of PFS and OS [69]. Beyond HER2- and TROP-2-directed strategies, additional ADC targets are emerging in EC. Early BEHOLD-1 data support the development of the B7-H4-directed ADC mocertatug rezetecan/GSK5733584 in recurrent or advanced EC and platinum-resistant OC [70]. Rinatabart sesutecan (Rina-S), a next-generation FRα-directed ADC, is also being evaluated in recurrent or progressive EC after prior platinum and PD-1/PD-L1 therapy (RAINFOL-03), highlighting the expansion of ADC development beyond HER2 alone.

### 3.5. Emerging Combinations and Ongoing Trials

Beyond established first-line and salvage regimens, several investigational strategies are addressing three major clinical questions in EC: whether immunotherapy should move into earlier curative-intent settings, how to improve immune responsiveness in pMMR/MSS disease, and how to treat patients after prior PD-1/PD-L1 exposure.

Neoadjuvant PD-1 blockade is also emerging as a strategy for surgically resectable dMMR/MSI-H EC. Neoadjuvant pembrolizumab has demonstrated safety and immunologic activity in dMMR EC [71]. The NIVEC trial is evaluating induction nivolumab in surgically resectable dMMR EC, reflecting growing interest in whether short-course preoperative ICI may inform future de-escalation strategies [72]. However, prospective validation is needed before further clinical adoption.

In the adjuvant, curative-intent setting, KEYNOTE-B21/ENGOT-en11/GOG-3053 did not show a disease-free survival (DFS) benefit in the overall EC population, but a prespecified dMMR subgroup analysis suggested a clinically meaningful DFS improvement (HR, 0.31; 95% CI, 0.14–0.69) [73,74]. These findings support further evaluation of biomarker-selected adjuvant immunotherapy and reinforce the importance of molecular testing at diagnosis. For pMMR/MSS disease, ongoing work is testing whether rational combinations can increase immunotherapy sensitivity. Studies such as RUBY Part 2, MITO END-3, and NRG-GY035 are evaluating whether PARP inhibition, antiangiogenic therapy, or other rational combinations can enhance immunotherapy sensitivity [75,76,77,78]. In parallel, emerging checkpoint targets are also under evaluation. LAG-3 is frequently co-expressed with PD-1 on tumor-infiltrating lymphocytes (TILs), particularly in dMMR tumors, supporting the rationale for LAG-3-directed combinations [79,80]. Early trials are also evaluating alternative checkpoint strategies, including anti-TIM-3 approaches [81], etigilimab (anti-TIGIT antibody) plus nivolumab in ACTIVATE trial [82], and NRG-GY025, which is testing nivolumab with low-dose ipilimumab (a CTLA-4 inhibitor) versus nivolumab alone in previously PD-1-exposed MSI-H/dMMR EC [83]. Together, these studies are addressing mechanisms of immune resistance and the potential value of alternative checkpoint blockade, supporting further evaluation of non-PD-1 checkpoint pathways in EC.

As frontline chemoimmunotherapy becomes increasingly common, treatment sequencing is becoming a central clinical challenge. KEYNOTE-C93 and DO-MENICA are directly comparing frontline PD-1 monotherapy with carboplatin and paclitaxel, testing whether chemotherapy can be deferred in this biomarker-defined population. The AFT-50/EndoMAP platform trial uses genomic screening to assign patients with recurrent or persistent EC to biomarker-matched cohorts, including atezolizumab with talazoparib, ipatasertib, trastuzumab emtansine, tiragolumab, or bevacizumab, as well as non-atezolizumab targeted therapy arms [84]. This design enables evaluation of IO-anchored versus non-IO strategies within a biomarker-driven platform. Together, these studies help to clarify how to personalize immunotherapy selection, maintenance, and sequencing across molecular subgroups, with universal molecular testing for POLE, MMR/MSI, p53, and HER2 remaining central to treatment selection.

### 3.6. Toxicities

While the combination of conventional chemotherapy with immuno-oncology (IO) allows for enhanced synergy of treatment response, additive side effects must also be carefully considered. For the most part, adverse effects from IO do not overlap with those commonly seen with cytotoxic chemotherapy (CTX). In particular, toxicities related to bone marrow suppression from CTX are not generally compounded with the addition of IO [85], and thus the safety profiles of combined regimens are related to the unique class of drugs themselves. Immune-related adverse effects (irAEs) from ICIs have been well-characterized, and guidelines have been established for early diagnosis and treatment [86]. Much of the clinical experience with irAEs from ICIs has been derived from the treatment of non-gynecologic solid tumors. Cutaneous irAEs have been reported in up to 40% of patients but rarely result in grade 3–4 toxicity that leads to cessation of therapy [86,87]. Endocrinopathy in the form of hypothyroidism or hypopituitarism may necessitate interruption of therapy with implementation of steroid therapy, but early detection of endocrine dysfunction from bloodwork prior to presentation of overt symptomatology can often mitigate these symptoms before they start [86,88]. In contrast, gastrointestinal irAEs, including nausea, vomiting and diarrhea, can be equally as common as endocrinopathies but are infrequently preceded by abnormal laboratory findings.

The emergence of ADCs as the most recent class of drug to be introduced in the treatment of gynecologic cancer has come with additional toxicity related to its targeted mechanism of action. Antigens on the eye offer collateral targets for the toxic payload from ADCs that result in significant adverse effects such as keratoconjunctivitis, dry eye, and resultant changes in visual acuity [89,90,91]. However, not all ADCs portend the same ocular toxicity. Among them, T-DXd used in HER2 positive tumors does not carry the same ocular toxicity boxed warning as others described below [92,93].

## 4. Cervical Cancer

In addition to molecularly guided immunotherapy in EC, cervical cancer provides a distinct model of immune-directed treatment shaped by HPV-associated tumor biology, with applications across locally advanced and recurrent/metastatic disease. PD-1 blockade, especially pembrolizumab, has become an increasingly important component of cervical cancer care, and PD-L1 expression should be assessed in all cervical tumors, along with additional biomarker testing to guide clinical decision-making and therapy selection [94]. The landscape has also broadened beyond single agents to include combination strategies with chemoradiotherapy, chemotherapy with or without bevacizumab, dual-checkpoint strategies, antibody–drug conjugates, and therapeutic vaccines.

### 4.1. Immunologic Rationale and Biomarker Framework

Cervical cancer is predominantly driven by high-risk human papillomavirus (HPV) infection, which provides a biologic rationale for immune-directed treatment strategies. HPV-derived oncoproteins E6 and E7 are tumor-associated viral antigens that can be recognized by the immune system, supporting the development of both immune checkpoint blockade and therapeutic vaccination strategies [95]. PD-L1 expression, assessed by combined positive score (CPS), is the most established actionable biomarker for ICI selection in cervical cancer, particularly in the recurrent or metastatic setting, where PD-L1 CPS ≥1 guides pembrolizumab-based therapy [9,30]. In advanced or recurrent disease, broader biomarker testing may identify additional biomarker-directed, tumor-agnostic treatment options based on MSI-H/dMMR, TMB-H, and HER2 positive IHC 3+ status [96]. Tissue factor expression, the target of tisotumab vedotin, is frequently expressed in cervical cancer; however, tissue factor testing does not currently serve as a predictive cutoff for treatment eligibility.

### 4.2. Locally Advanced Disease

In locally advanced cervical cancer, KEYNOTE-A18 (ENGOT-cx11/GOG-3047) established pembrolizumab plus chemoradiotherapy, followed by pembrolizumab maintenance, as a new treatment standard. The regimen improved both PFS and OS, with benefit primarily driven by patients with FIGO 2014 stage III–IVA disease [97,98]. Based on these data, the FDA approved pembrolizumab with chemoradiotherapy in January 2024 for FIGO 2014 stage III–IVA cervical cancer. In contrast, the CALLA trial with durvalumab (anti-PD-L1) plus chemoradiotherapy did not significantly improve PFS [99]. These divergent results suggest that the benefit of adding ICI to chemoradiotherapy may depend on trial population, treatment timing, maintenance strategy, agent selection, and biomarker context. Early-phase studies have further demonstrated the feasibility and safety of combining PD-(L)1 inhibitors with pelvic chemoradiation, including nivolumab with chemoradiotherapy in the NICOL trial and atezolizumab given either neoadjuvantly or concurrently with chemoradiotherapy in NRG-GY017 [100,101].

Taken together, these data indicate that immunotherapy is moving into curative-intent treatment for selected patients with locally advanced cervical cancer, but the benefit of adding ICI to chemoradiation is not uniform across trials. Future studies are needed to refine patient selection and optimal treatment sequencing.

### 4.3. Recurrent or Metastatic Disease

**Figure 2:**
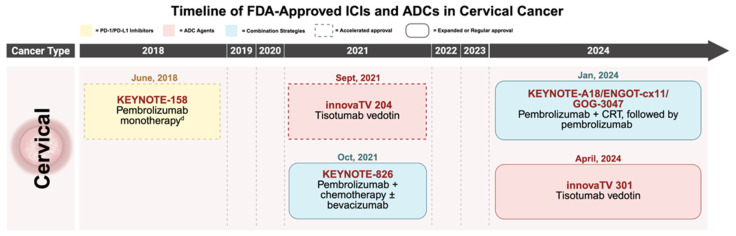
Timeline of FDA approvals of ICIs and ADCs in cervical cancer. Abbreviations: ADC, antibody–drug conjugate; CRT, chemoradiation therapy; FDA, U.S. Food and Drug Administration; ICIs, immune checkpoint inhibitors. ^d^ Pembrolizumab monotherapy for PD-L1 CPS ≥1 recurrent or metastatic CC received accelerated approval in June 2018 and regular approval in October 2021.

In recurrent or metastatic cervical cancer, the role of immune checkpoint blockade has evolved from salvage monotherapy to first-line combination treatment. Pembrolizumab initially received accelerated FDA approval in 2018 for PD-L1 CPS ≥1, recurrent or metastatic cervical cancer after progression on or after chemotherapy, based on KEYNOTE-158 [29] (Figure 2). In addition to its cervical cancer-specific indication, pembrolizumab is also listed in the NCCN Guidelines as a biomarker-directed option for recurrent or metastatic cervical cancer with MSI-H/dMMR or TMB-H status, reflecting its broader tumor-agnostic activity.

Subsequent phase III trials have established ICIs as part of first-line and subsequent treatment strategies for persistent, recurrent, or metastatic cervical cancer. In KEYNOTE-826, the addition of pembrolizumab to platinum-based chemotherapy, with or without bevacizumab, improved survival outcomes compared with placebo plus chemotherapy, with consistent benefit across clinically relevant subgroups, including by bevacizumab use [9,30,102,103]. BEATcc further supported the strategy of adding atezolizumab to the platinum–taxane–bevacizumab backbone, demonstrating significantly improved PFS and OS compared with bevacizumab plus chemotherapy alone [104]. Although atezolizumab does not currently have a U.S. FDA-approved cervical cancer indication, this regimen is listed in the NCCN Guidelines as a first-line option for metastatic, persistent, or recurrent cervical cancer [105].

In the second-line or later setting, EMPOWER-Cervical 1/GOG-3016/ENGOT-cx9 demonstrated that cemiplimab improved OS compared with investigator’s choice single-agent chemotherapy after progression on first-line platinum-based therapy [106], with the benefit observed regardless of PD-L1 expression [107]. Although cemiplimab does not currently have a U.S. FDA-approved cervical cancer indication, these findings support the clinical activity of PD-1 blockade beyond the first-line setting.

Collectively, these studies establish ICI as an important component of systemic therapy for recurrent/metastatic cervical cancer. In clinical practice, PD-L1 status, prior therapy, bevacizumab eligibility, and regulatory indication all influence treatment selection.

### 4.4. Dual Checkpoint Blockade

Dual checkpoint blockade represents an emerging strategy to enhance antitumor immune activity in cervical cancer by targeting the inhibitory PD-1 and CTLA-4 pathways. In CheckMate 358, nivolumab plus ipilimumab demonstrated numerically higher response rates than nivolumab monotherapy in recurrent or metastatic cervical cancer, supporting further evaluation of chemotherapy-free dual immunotherapy approaches [22]. Additional early-phase studies, including balstilimab (anti-PD-1) plus zalifrelimab (anti-CTLA-4) have also shown clinical activity after prior platinum-based therapy [108]. More recently, COMPASSION-16 (NCT04982237) demonstrated that cadonilimab, a bispecific antibody targeting both PD-1 and CTLA-4, significantly improved PFS and OS when combined with first-line platinum-based chemotherapy, with or without bevacizumab, compared with chemotherapy alone [109]. Collectively, these findings support dual checkpoint blockade as a promising strategy, although its optimal role relative to PD-1/PD-L1-based chemoimmunotherapy and ADC-based approaches remains to be defined.

### 4.5. Antibody–Drug Conjugates (ADCs)

ADCs have expanded treatment options for recurrent or metastatic cervical cancer by providing target-directed cytotoxic therapy beyond conventional chemotherapy and immune checkpoint blockade. Tisotumab vedotin is a tissue factor-directed ADC approved by the FDA for recurrent or metastatic cervical cancer with disease progression on or after chemotherapy [110]. In the phase III innovaTV 301 trial, tisotumab vedotin significantly improved OS, PFS, and ORR compared with investigator’s choice chemotherapy in the second- or third-line setting [53].

Another ADC relevant to cervical cancer is trastuzumab deruxtecan. As discussed in the endometrial cancer section, DESTINY-PanTumor02 was a pan-tumor study rather than a cancer-specific trial, but cervical cancer was included among the evaluated tumor cohorts. Consistent with this biomarker-directed approach, the NCCN Guidelines list fam-trastuzumab deruxtecan-nxki as a “Useful in Certain Circumstances” option for HER2-positive cervical cancer, including tumors with HER2 IHC 3+ or 2+ expression [105].

Beyond monotherapy, tisotumab vedotin-based combinations have been evaluated in innovaTV 205/GOG-3024/ENGOT-cx8 study. This open-label study evaluated tisotumab vedotin in combination with bevacizumab, carboplatin, or pembrolizumab in recurrent or metastatic cervical cancer [111]. These combinations demonstrated manageable safety and encouraging antitumor activity, supporting further evaluation of tisotumab vedotin-based combination strategies. Although pembrolizumab plus tisotumab vedotin is not FDA-approved as a combination regimen, it is listed in the NCCN Guidelines as a “Useful in Certain Circumstances” second-line or subsequent therapy option for PD-L1-positive, IO-naive recurrent or metastatic cervical cancer [105].

In addition, emerging TROP-2-directed ADCs may further broaden ADC development in cervical cancer. Preclinical studies have shown sensitivity of TROP-2-overexpressing cervical cancer models to sacituzumab govitecan and datopotamab deruxtecan [112,113]. Recently, single-agent sacituzumab govitecan demonstrated antitumor activity in previously treated recurrent or metastatic cervical cancer, including patients previously exposed to immunotherapy, supporting continued clinical evaluation of TROP-2-directed ADCs in cervical cancer [114]. Clinically, ADCs complement rather than replace ICI-based strategies in cervical cancer. Ongoing studies will need to clarify whether ADCs are best used sequentially after ICI-based treatment or concurrently with immunotherapy, chemotherapy, or antiangiogenic agents.

### 4.6. Therapeutic Vaccination

Therapeutic vaccination represents an emerging immunologic strategy in HPV-driven cervical cancer. In the single-arm phase 2a study, VB10.16, a DNA-based HPV16 E6/E7 vaccine, combined with atezolizumab demonstrated an ORR of 19.1% with durable responses and manageable safety in persistent, recurrent, or metastatic HPV16-positive cervical cancer, supporting further evaluation of vaccine and ICI combination strategies [115].

Beyond therapeutic vaccines, HPV-directed cellular therapies are being investigated for HPV-associated cervical cancer. Early clinical studies of HPV-reactive TILs and HPV16 E7-directed T-cell receptor (TCR)-engineered T cells have demonstrated clinical activity in metastatic HPV-associated epithelial cancers, including cervical cancer [116,117]. Preclinical studies have also supported the feasibility of HPV16 E6- or E7-directed TCR-engineered T-cell approaches, providing a rationale for ongoing cellular therapy development in selected HPV-associated tumors [118,119].

Together, therapeutic vaccines and HPV-directed cellular therapies extend the cervical cancer immunotherapy landscape beyond checkpoint blockade, although their optimal integration with ICIs, ADCs, chemotherapy, and chemoradiotherapy remains investigational.

### 4.7. Toxicities

As described earlier, ADCs can carry additional concern of ocular toxicity. The widespread adoption of tisotumab vedotin as a first-line salvage treatment for recurrent or metastatic cervical cancer has required judicious clinical surveillance for manifestations of ocular toxicity given its FDA boxed warning [92,105]. Dose reduction and potential discontinuation of treatment are most commonly a result of the aforementioned ocular complications [120].

## 5. Ovarian Cancer

Unlike endometrial and cervical cancers, in which immune-directed therapy has become increasingly established, ovarian cancer (OC) has historically been more challenging for immunotherapy because of its immune-cold tumor microenvironment, substantial tumor heterogeneity, and multiple overlapping resistance mechanisms [121]. Nevertheless, accumulating biologic and clinical evidence has refined understanding of immune responsiveness in OC and has supported rational combination strategies, biomarker-enriched trial designs, and ADC development.

### 5.1. Immune Biology and Rationale for Immunotherapy

The rationale for immune checkpoint inhibition in OC is supported by evidence of immune recognition within the tumor microenvironment. Tumor-infiltrating lymphocytes, particularly intraepithelial CD8+ T cells, have been associated with improved prognosis in epithelial OC [122]. These findings suggest that pre-existing antitumor immunity exists in a subset of ovarian cancers and may be therapeutically reactivated.

However, OC also contains multiple mechanisms of immune escape that limit response to checkpoint blockade. Tumor-intrinsic features, including dMMR, high TMB, and increased neoantigen load, may enhance immunogenicity in selected tumors, whereas the predictive value of homologous recombination deficiency (HRD) for ICI benefit remains inconsistent. Immune suppression within the tumor microenvironment is mediated by PD-1/PD-L1 signaling, regulatory T cells, tumor-associated macrophages, VEGF-driven abnormal vasculature, hypoxia, and metabolic suppression. The coexistence of immune-activating and immune-suppressive forces likely explains why ICIs have shown inconsistent activity in OC and why unselected populations have generally derived limited benefit from ICI-based approaches [121,123].

### 5.2. Immune Checkpoint Inhibitor-Based Strategies in Ovarian Cancer

#### 5.2.1. ICI Monotherapy

Single-agent immune checkpoint blockade has demonstrated limited efficacy in unselected recurrent OC. Early studies of pembrolizumab, nivolumab, avelumab, and atezolizumab generally showed modest ORR, typically around or below 15%, with short median PFS [124,125,126,127,128,129]. KEYNOTE-028 and KEYNOTE-100 demonstrated low response rates with pembrolizumab, although numerically higher responses were observed in patients with higher PD-L1 CPS [124,125]. The negative NINJA trial further supports this pattern, showing that nivolumab did not improve OS and was associated with inferior PFS compared with chemotherapy in platinum-resistant OC, arguing against replacement of cytotoxic chemotherapy with single-agent PD-1 blockade in unselected patients [127]. Collectively, these studies indicate that single-agent PD-1/PD-L1 blockade is insufficient for most patients with advanced or recurrent OC. A recent meta-analysis of randomized trials also found that anti-PD-1/PD-L1-based strategies did not consistently improve PFS in advanced OC across disease settings (first-line or recurrent settings) or biomarker-defined subgroups [130].

#### 5.2.2. ICI Plus Chemotherapy

In the frontline setting, adding ICIs to standard chemotherapy has not consistently improved outcomes. JAVELIN Ovarian 100 evaluated avelumab combined with carboplatin and paclitaxel followed by avelumab maintenance, but the trial was terminated for futility and did not demonstrate a PFS benefit [131]. IMagyn050 evaluated atezolizumab added to carboplatin, paclitaxel, and bevacizumab in newly diagnosed stage III or IV OC but failed to improve PFS, and final analyses did not show an OS benefit [132,133]. Biomarker analyses did not identify clear predictive value for BRCA mutation status, HRD, or PD-L1 expression, although post hoc analyses suggested possible benefits in selected poor-prognosis subgroups [134,135].

**Figure 3:**
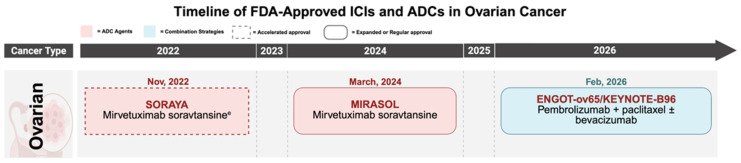
Timeline of FDA approvals of ICIs and ADCs in ovarian cancer. Abbreviations: ADC, antibody–drug conjugate; FDA, U.S. Food and Drug Administration; ICIs, immune checkpoint inhibitors. ^e^ PROC refers to platinum-resistant epithelial ovarian, fallopian tube, or primary peritoneal cancer.

In contrast, the platinum-resistant setting has recently produced a clinically meaningful positive result. ENGOT-ov65/KEYNOTE-B96 evaluated pembrolizumab plus weekly paclitaxel, with or without bevacizumab, versus placebo plus weekly paclitaxel, with or without bevacizumab, in platinum-resistant recurrent ovarian cancer (PROC) [136], supporting FDA approval in this biomarker-selected setting (Figure 3). By contrast, AGO-OVAR 2.29/ENGOT-ov34 did not improve outcomes when atezolizumab was added to bevacizumab and non-platinum chemotherapy in recurrent OC, suggesting that ICI benefit in OC may depend on the checkpoint inhibitor, chemotherapy backbone, treatment setting, and biomarker-selected population [137]. Together, these findings suggest that ICI benefit in OC depends on disease setting, biomarker selection, chemotherapy backbone, and checkpoint inhibitor context rather than chemotherapy combination alone.

#### 5.2.3. ICI Plus PARP Inhibitors and Antiangiogenic Combinations

Frontline maintenance strategies incorporating ICIs with PARP inhibitors and antiangiogenic agents have produced mixed results, underscoring the complexity of immune modulation in OC. DUO-O, KEYLYNK-001, and FIRST reported PFS improvements with ICI-containing combination strategies; however, interpretation is limited by differences in trial design, maintenance backbone, biomarker-defined populations, and the absence of PARP inhibitor-only control arms in some studies [138,139,140]. In contrast, ATHENA-COMBO showed no benefit, and possible detriment, when nivolumab was added to rucaparib maintenance compared with rucaparib alone, emphasizing that ICI plus PARP inhibitor combinations are not uniformly beneficial [141,142].

In recurrent disease, results have also been inconsistent. TOPACIO/KEYNOTE-162 demonstrated modest activity with niraparib plus pembrolizumab in recurrent PROC regardless of BRCA status [143], whereas ANITA and ATALANTE did not meet their primary PFS objectives with atezolizumab-containing combinations [144,145]. These mixed results suggest that ICI activity in OC depends heavily on treatment context, immune microenvironment characteristics, and biomarker enrichment rather than checkpoint blockade alone.

Overall, negative and mixed ICI trials in OC should not be viewed simply as failed studies, but as evidence that ICI benefit depends on the appropriate immune context, disease setting, and biomarker strategy. Biologically, these results likely reflect the immune-cold and heterogeneous nature of ovarian cancers, including limited baseline T-cell infiltration, T-cell exhaustion, and inconsistent predictive value of PD-L1, BRCA, or HRD status for ICI benefit. Methodologically, many trials enrolled broad populations without strong immune biomarker enrichment, used different chemotherapy or maintenance backbones, and evaluated ICIs in different disease settings. Moreover, many biomarker-based subgroup findings were exploratory or post hoc, limiting their ability to guide definitive patient selection. These findings argue against unselected use of ICIs in OC and support future trials focused on biomarker-enriched populations, rational sequencing, and resistance-guided combinations.

#### 5.2.4. Histology-Specific Considerations: Clear Cell Carcinoma

Among ovarian cancer histologic subtypes, clear cell carcinoma may represent a more immunoresponsive population. Early-phase and nonrandomized studies, including LARA with pembrolizumab plus lenvatinib, and MoST-CIRCUIT, which studied dual PD-1/CTLA-4 blockade with nivolumab plus ipilimumab, have reported ORRs of 40% to approximately 55% in gynecologic or ovarian clear cell carcinoma, supporting further evaluation of histology-enriched ICI strategies [20,146]. Although these data remain early and require prospective validation, clear cell carcinoma illustrates how histology-enriched trial design may identify OC subgroups with greater immunotherapy sensitivity than unselected high-grade serous populations.

### 5.3. Antibody–Drug Conjugates in Ovarian Cancer

Given the limited success of ICI-based strategies in unselected OC populations, ADC-based approaches have become increasingly important. By enabling targeted cytotoxic delivery, ADCs may directly induce tumor cell death while also promoting immunogenic cell death, antigen release, and tumor microenvironment remodeling, thereby providing a rationale for future ADC-ICI combination strategies.

#### 5.3.1. FRα-Directed ADC Therapy

Mirvetuximab soravtansine is an FRα-directed ADC and the most clinically validated ADC in OC. After initial accelerated FDA approval based on SORAYA [52,147], the confirmatory phase III MIRASOL trial demonstrated improved ORR, PFS and OS compared with investigator’s choice chemotherapy in FRα-high PROC [11], supporting regular FDA approval in this setting [148]. The PICCOLO trial further suggests potential activity beyond the platinum-resistant setting, including later-line platinum-sensitive disease [149]. Clinically, the use of mirvetuximab makes FRα assessment directly relevant to treatment selection in OC and highlights the importance of matching ADC therapy to tumor target expression.

#### 5.3.2. HER2-Directed ADC Therapy

**Figure 4:**
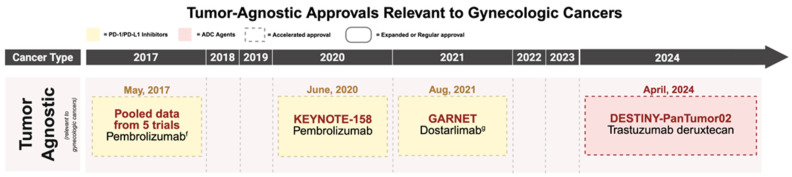
Timeline of tumor-agnostic FDA approvals relevant to gynecologic cancers. Abbreviations: ADC, antibody–drug conjugate; FDA, U.S. Food and Drug Administration; ICIs, immune checkpoint inhibitors. ^f^ The MSI-H/dMMR tumor-agnostic pembrolizumab indication was initially granted accelerated approval in 2017 as the FDA’s first tissue/site-agnostic approval. It was converted to regular approval in March 2023, for adult and pediatric patients with un-resectable or metastatic MSI-H/dMMR solid tumors that progressed following prior treatment and for whom no satisfactory alternative treatment options are available, based on KEYNOTE-158, KEYNOTE-164, and KEYNOTE-051. ^g^ Dostarlimab received tumor-agnostic accelerated approval on August 17, 2021, for adult patients with dMMR recurrent or advanced solid tumors that progressed on or following prior treatment and who have no satisfactory alternative treatment options. All figures were created with BioRenderHsieh, T. Y. J. (2026).

Trastuzumab deruxtecan is a HER2-directed ADC with tumor-agnostic FDA accelerated approval for adults with unresectable or metastatic HER2-positive IHC 3+ solid tumors after prior systemic therapy and when no satisfactory alternative treatment options are available [150]. This approval was supported in part by DESTINY-PanTumor02, which included OC cohorts and demonstrated clinically meaningful activity across HER2-expressing solid tumors, with the greatest benefit in HER2 IHC 3+ tumors [12,151]. In ovarian cancer, the NCCN Guidelines list trastuzumab deruxtecan more broadly as a biomarker-directed option for selected HER2-positive tumors, including tumors with HER2 IHC 3+ or 2+ expression [152] (Figure 4).

#### 5.3.3. Emerging ADC Targets Beyond FRα and HER2

Beyond FRα and HER2, the ovarian cancer ADC landscape is expanding to include B7-H4, NaPi2b, TROP-2, c-Met, and next-generation FRα-directed constructs. B7-H4 is an emerging ADC target in gynecologic cancers, and early BEHOLD-1 data support B7-H4-directed ADC development with mocertatug rezetecan in PROC and recurrent or advanced EC [70]. Next-generation FRα-directed ADCs, including rinatabart sesutecan (Rina-S) and luveltamab tazevibulin (STRO-002), are also under active investigation, with preclinical data supporting activity in FRα-expressing ovarian and endometrial tumors [153]. TROP-2-directed ADCs, including datopotamab deruxtecan and sacituzumab govitecan-based strategies, and c-Met-directed ADCs, such as telisotuzumab adizutecan, are also under early clinical or translational evaluation in gynecologic cancers [154,155,156]. However, the UPLIFT trial with the NaPi2b-directed ADC upifitamab rilsodotin did not meet the primary endpoint in PROC patients and thus the trial development has dicontinued [157,158]. Future ADC development in OC will require optimized biomarker thresholds, standardized assays, evaluation of antigen heterogeneity, toxicity monitoring, and strategies for sequencing after prior ADC exposure.

### 5.4. Toxicities

Both IO and ADC have come to the forefront of treatment in platinum-resistant recurrent OC. The recent indication for use of pembrolizumab in PD-L1-expressing recurrent OC, in combination with weekly paclitaxel based on EN-GOT-ov65/KEYNOTE-B96, showed no additive bone marrow toxicity compared with controls [136]. These safety data, along with RUBY and NRG-GY018 trials in EC and KEYNOTE-826 in cervical cancer, have endorsed the safety of ICIs in combination with CTX [7,9,85]. In the SORAYA trial, the incidence of ocular complications was over 50% in patients with recurrent OC. However, only 6% developed severe keratinopathy which would obligate discontinuation of therapy [52].

## 6. Challenges and Future Directions

Despite rapid progress in ICIs and ADCs, several major challenges remain across gynecologic oncology. The central question is no longer whether these therapies can be active, but how to identify patients most likely to benefit, how to sequence therapies after prior exposure, and how to balance efficacy with cumulative toxicity. Future progress will depend on biomarker-enriched trial designs, rational combinations, longitudinal immune profiling, improved understanding of resistance mechanisms, and incorporation of patient-centered outcomes.

### 6.1. Resistance Mechanisms

Resistance to ICIs and ADCs remains a major barrier to durable benefits in gynecologic cancers. ICI resistance may be primary or acquired and can result from impaired antigen presentation, defective interferon signaling, T-cell exhaustion, myeloid-mediated immune suppression, regulatory T-cell enrichment, angiogenesis, hypoxia, and metabolic suppression within the tumor microenvironment [159,160]. These mechanisms are particularly relevant in ovarian cancer, where tumor heterogeneity contributes to the limited activity of single-agent checkpoint blockade. In a landmark study of advanced epithelial OC, CD3-positive intratumoral T cells were detected in only 54.8% of tumors, supporting the concept that many ovarian cancers may lack the baseline T-cell infiltration needed for effective PD-1/PD-L1 reinvigoration [161]. Even in immune-inflamed tumors, co-expression of additional inhibitory receptors, such as LAG-3, TIM-3, or CTLA-4, may contribute to adaptive resistance and provides a rationale for multi-checkpoint strategies [162].

ADC resistance can occur through heterogeneous or low target-antigen expression, antigen loss after treatment exposure, altered ADC internalization or intracellular trafficking, impaired lysosomal processing, drug efflux, and payload resistance [163,164]. These mechanisms have direct implications for future trial design. Rather than relying only on baseline biomarkers, future studies should incorporate serial biomarker testing, circulating tumor DNA, and immune profiling to guide ICI rechallenge, ADC sequencing, target switching, and rational resistance-directed combinations.

### 6.2. Biomarker Limitations

Although biomarkers play an essential role in ICI and ADC selection, current biomarkers remain incomplete predictors of benefit. In EC, MMR/MSI status is one of the most clinically informative biomarkers for ICI responsiveness, but discordance between MMR IHC and MSI-based testing, heterogeneous MMR protein expression, and assay-related variability can complicate interpretation [165]. Thus, comprehensive molecular assessment rather than reliance on a single biomarker is highlighted [166]. PD-L1 expression is also not a perfect biomarker because its interpretation may vary by tumor type, stage, assay platform, scoring method, and treatment exposure [167,168]. In cervical cancer, although PD-L1 CPS is clinically useful for pembrolizumab-based treatment selection, its predictive value is limited by threshold-dependent scoring, suboptimal negative predictive value, and interobserver variability [169].

ADC biomarker interpretation is also limited. In EC, discordance between sequencing-based and IHC-based assessment may occur when HER2 amplification is heterogeneous, tumor cellularity is limited, or results fall near predefined positivity thresholds, potentially altering eligibility for HER2-directed therapy [170]. Future biomarker strategies should incorporate spatial heterogeneity, target-antigen expression, circulating tumor DNA dynamics, and longitudinal biomarker changes after therapy to improve patient selection and guide sequencing or combination treatment.

### 6.3. Sequencing and Rational Combinations

As ICIs and ADCs move into earlier lines of therapy, optimal sequencing after prior exposure is becoming increasingly important. In EC, key questions include whether chemotherapy can be safely deferred in highly immunogenic dMMR/MSI-H disease and how to treat patients after prior PD-1 or PD-L1 marker-directed therapy. In cervical cancer, future studies should clarify how best to sequence ICIs, ADCs, and therapeutic vaccine strategies as these treatments move across earlier and later lines of therapy. In OC, where ICI benefit has been less consistent, treatment sequencing will require more precise integration of platinum sensitivity, immune biomarkers, ADC target expression, and prior PARP inhibitor or antiangiogenic exposure.

Rational combinations remain essential but need to be developed carefully. ADCs may enhance immune engagement through cytotoxic tumor killing, antigen release, and immunogenic cell death, providing a biologic rationale for ADC–ICI combinations [171]. However, not all combinations are synergistic, and overlapping toxicities may limit clinical utility. Pneumonitis or interstitial lung disease is particularly relevant for trastuzumab deruxtecan, whereas tisotumab vedotin has distinct toxicities, including ocular toxicity and peripheral neuropathy [92,93]. These concerns support careful toxicity monitoring, biomarker-driven enrichment, early stopping rules, and studies comparing concurrent versus sequential approaches [172].

### 6.4. Toxicity Management

As ICIs and ADCs move into earlier lines and combination regimens, proactive toxicity management is essential. ICI-related toxicities can involve nearly any organ system and require patient education, early recognition and work-up, ICI treatment interruption, corticosteroids or other immunosuppression when indicated, and careful rechallenge decisions [86]. In gynecologic cancers, ICI-based therapy has improved efficacy outcomes without a major increase in overall treatment-related adverse events, but immune-related adverse events and treatment discontinuation are approximately 3-fold higher with ICI-containing regimens [173].

ADC toxicities require similarly proactive but agent-specific management. Trastuzumab deruxtecan-associated pneumonitis or interstitial lung disease requires symptom surveillance, early imaging and diagnostic evaluation, treatment interruption or discontinuation according to severity, prompt corticosteroid treatment when clinically indicated, and multidisciplinary management [174,175]. Ocular toxicity is particularly relevant for tisotumab vedotin and mirvetuximab soravtansine, requiring baseline and serial eye examinations, prophylactic eye care, patient education, prompt referral for new symptoms, and dose modification when needed [89,90,91]. Future trials should define toxicity management algorithms prospectively, report dose reductions and discontinuation rates, and clarify when concurrent versus sequential strategies provide the best balance of efficacy and tolerability.

### 6.5. Patient-Reported Outcomes and Quality of Life

As ICI- and ADC-based strategies extend survival and move into maintenance or earlier-line settings, patient-reported outcomes (PROs) are increasingly important for assessing net clinical benefit. PFS, OS, and ORR do not fully capture symptom burden, chronic immune-related toxicity, treatment access, financial toxicity, or functional recovery. Across solid tumors, ICIs have been associated with delayed clinical deterioration and better preservation of several quality-of-life (QoL) measures compared with chemotherapy, although these data are not specific to gynecologic malignancies [176]. In gynecologic cancers, a systematic review and meta-analysis found that ICI-based therapy did not significantly change mean QoL scores compared with controls, but was associated with longer time to definitive QoL deterioration [173].

Disease-specific PRO data further support incorporating patient-centered endpoints into gynecologic oncology trials. Recent patient-reported outcome analyses across KEYNOTE-826, RUBY, and MIRASOL suggest that selected ICI- and ADC-based strategies can maintain health-related QoL despite treatment intensification or use in recurrent disease settings [177,178,179]. Future trials should prospectively incorporate validated disease-specific PRO instruments, time to symptom deterioration, treatment-related symptom scales, and patient-centered endpoints, particularly for maintenance therapy and ICI–ADC combinations.

Overall, the next phase of ICI and ADC development in gynecologic oncology should move beyond drug approval alone toward more precise treatment personalization. This will require integration of baseline and longitudinal biomarkers, post-progression resistance profiling, rational sequencing strategies, proactive toxicity management, and patient-centered outcome assessment to maximize durable benefit while minimizing ineffective treatment, cumulative toxicity, and quality-of-life burden.

## 7. Conclusions

ICIs and ADCs have reshaped the treatment landscape of gynecologic cancers, but their benefits vary substantially across tumor types and biomarker-defined populations. Future progress will depend on refining predictive biomarkers, optimizing treatment sequencing after prior ICI or ADC exposure, improving the safety of combination strategies, and designing trials that enrich for biologically responsive populations. Across ovarian, endometrial, and cervical cancers, the field is moving toward a more personalized therapeutic framework that integrates molecular classification, immune contexture, ADC target expression, and patient-specific clinical factors to guide treatment selection.

## Data Availability

No new data were created or analyzed in this study. Data sharing is not applicable to this article.
